# Supplementary material for: A bacterial display system for effective selection of protein-biotin ligase BirA variants with novel peptide specificity
Source: Sci Rep. 2019 Mar 11;9:4118. doi: 10.1038/s41598-019-40984-x (PMC6411976; doi:10.1038/s41598-019-40984-x)

Supplementary Information

A bacterial display system for effective selection of protein-biotin ligase BirA variants with novel peptide specificity

Jeff Granhøj, Henrik Dimke and Per Svenningsen*

Department of Molecular Medicine, University of Southern Denmark, Odense, Denmark

* Corresponding author: Dr. Per Svenningsen, Institute of Molecular Medicine, University of Southern Denmark, J.B. Winsloews vej 21.3, DK-5000 Odense C, Denmark.

Supplementary Figure 1

a

Nucleotide sequence of BirA-6xHis. The hexahistidine sequence is shown in green.

ATGAAGGATAACACCGTGCCACTGAAATTGATTGCCCTGTTAGCGAACGGTGAATTTC
ACTCTGGCGAGCAGTTGGGTGAAACGCTGGGAATGAGCCGGGCGGCTATTAATAAACA
CATTCAGACACTGCGTGACTGGGGCGTTGATGTCTTTACCGTTCCGGGTAAAGGATACA
GCCTGCCTGAGCCTATCCAGTTACTTAATGCTAAACAGATATTGGGTCAGCTGGATGGC
GGTAGTGTAGCCGTGCTGCCAGTGATTGACTCCACGAATCAGTACCTTCTTGATCGTAT
CGGAGAGCTTAAATCGGGCGATGCTTGCATTGCAGAATACCAGCAGGCTGGCCGTGGT
CGCCGGGGTCGGAAATGGTTTTCGCCTTTTGGCGCAAACTTATATTTGTCGATGTTCTG
GCGTCTGGAACAAGGCCCGGCGGCGATTGGTTTAAGTCTGGTTATCGGTATCGTG
ATGGCGGAAGTATTACGCAAGCTGGGTGCAGATAAAGTTCGTGTTAAATGGCCTAATG
ACCTCTATCTGCAGGATCGCAAGCTGGCAGGCATTCTGGTGGAGCTGACTGGCAAAAC
TGGCGATGCGGCGCAAATAGTCATTGGAGCCGGGATCAACATGGCAATGCGCCGTGTT
GAAGAGAGTGTCGTTAATCAGGGGTGGATCACGCTGCAGGAAGCGGGGATCAATCTCG
ATCGTAATACGTTGGCGGCCATGCTAATACGTGAATTACGTGCTGCGTTGGAACTCTTC
GAACAAGAAGGATTGGCACCTTATCTGTCGCGCTGGGAAAAGCTGGATAATTTTATTA
ATCGCCCAGTGAAACTTATCATTGGTGATAAAGAAATATTTGGCATTTCACGCGGAAT
AGACAAACAGGGGGCTTTATTACTTGAGCAGGATGGAATAATAAAACCCTGGATGGGC
GGTGAAATATCCCTGCGTAGTGCAGAAAAACATCATCATCATCATCATTGA

b

Nucleotide sequence of eCPX-AP (the AP sequence is shown in green)
ATGAAAAAAATCGCATGTCTGAGCGCACTGGCAGCAGTTCTGGCATTTACCGCAGGCA
CCAGCGTTGCCGGTGGTCAGAGCGGTCAGAGTGGTGATTATAACAAAAATCAGTATTA
CGGCATTACAGCCGGTCCGGCATATCGTATTAATGATTGGGCAAGCATTTATGGTGTTG
TGGGTGTTGGTTATGGTAAATTTCAGACCACCGAATATCCGACCTATAAACATGATACC
AGCGATTATGGTTTTAGCTATGGTGCAGGTCTGCAGTTTAATCCGATGGAAAATGTTGC
ACTGGATTTCAGCTATGAACAGAGCCGTATTCGTAGCGTTGATGTTGGCACCTGGATTC
TGAGCGTTGGTTATCGTTTTGGTAGCAAAAGCCGTCGTGCAACCAGCACCGTTACCGGT
GGTTATGCACAGAGTGATGCACAGGGTCAGATGAATAAAATGGGTGGCTTTAATCTGA
AATATCGCTACGAAGAAGATAATAGTCCGCTGGGTGTTATTGGTAGCTTTACCTATACC
GAAAAAAGCCGTACCGCAAGCGGTGCACGTCGTGCAGGCGGCCGCGGTCTGAATGAT
ATTTTTGAAGCACAGAAAATCGAGTGGCACGAGCTTAAGTAA

Supplementary Figure 2

Uncropped version of Figure 2a (top)

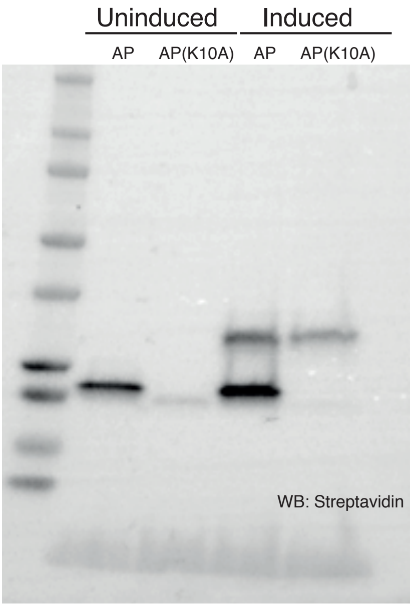

Supplementary Figure 3

Uncropped version of Figure 2a (bottom)

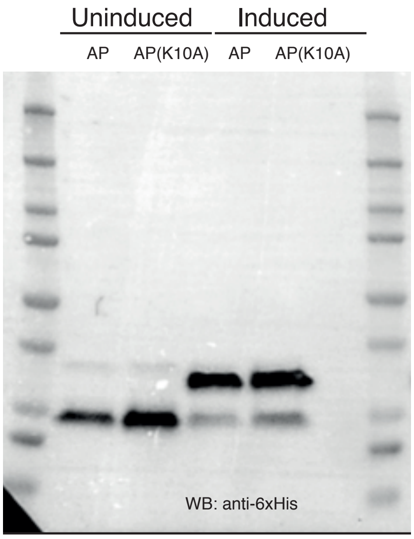

Supplementary Figure 4

Uncropped version of Figure 2c (top)

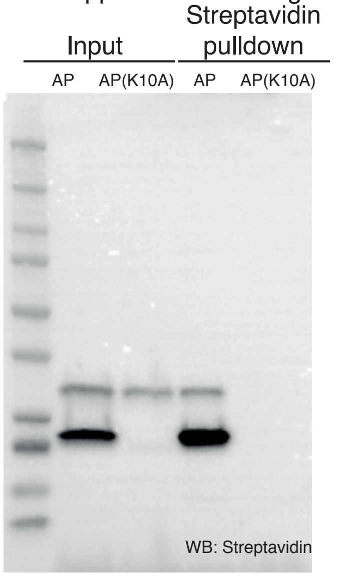

Supplementary Figure 5

Uncropped version of Figure 2c (bottom)

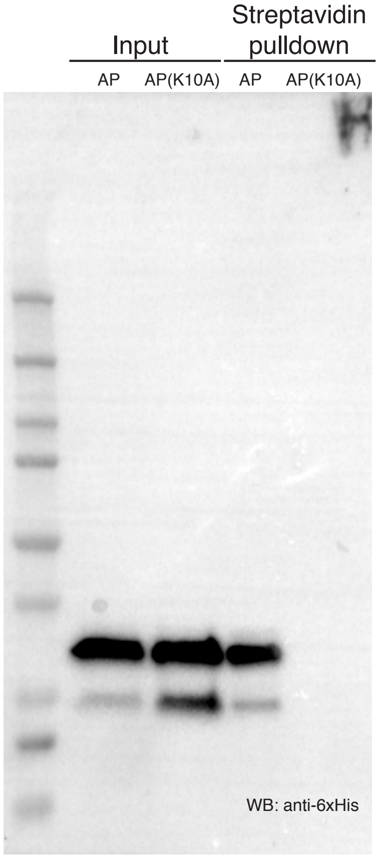

Supplementary Figure 6

Uncropped version of Figure 3a

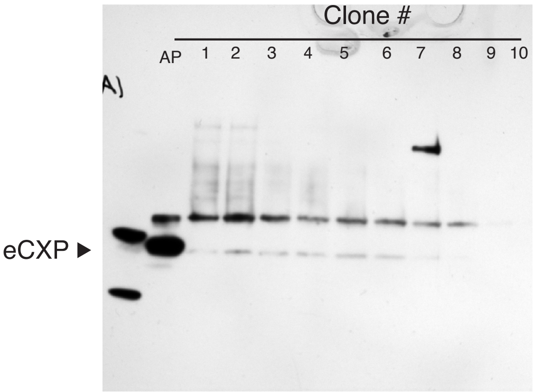

Supplementary Figure 7

Uncropped version of Figure 3b

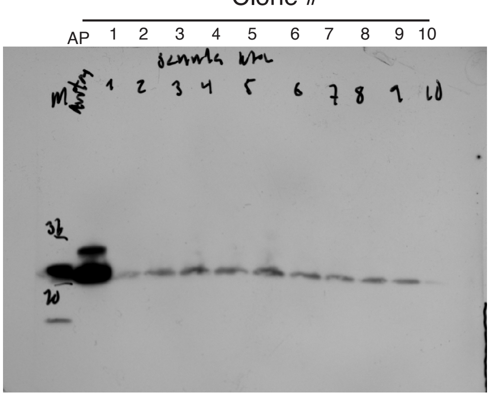

Supplementary Figure 8

Uncropped version of Figure 3c

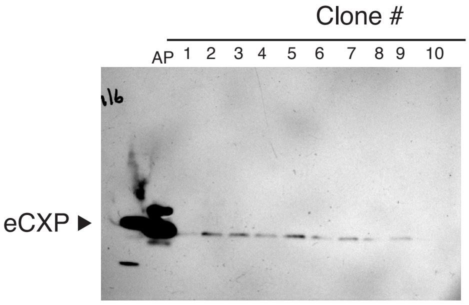

Supplementary Figure 9

Uncropped version of Figure 3d

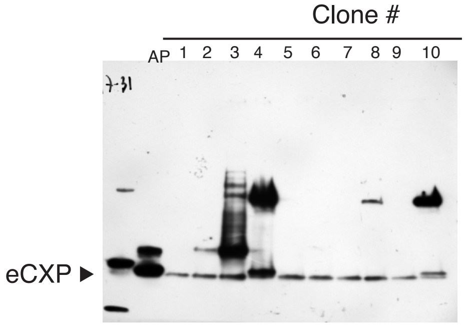

Supplementary Figure 10

(a) Alignment of BirA-6xHis and 10 isolated TagRFP K10 clones

```
CLUSTAL O(1.2.4) multiple sequence alignment


eCPX_TagRFP_K10_clone1_pTrcHisrev       TCAATGATGATGATGATGATGTTTTTCTACACTACGCAGGGATATTTCACCGCCCATCCA    60
eCPX_TagRFP_K10_clone2_pTrcHisrev       TCAATGATGATGATGATGATGTTTTTCTACACTACGCAGGGATATTTCACCGCCCATCCA    60
eCPX_TagRFP_K10_clone3_pTrcHisrev       TCAATGATGATGATGATGATGTTTTTCTACACTACGCAGGGATATTTCACCGCCCATCCA    60
eCPX_TagRFP_K10_clone4_pTrcHisrev       TCAATGATGATGATGATGATGTTTTTCTACACTACGCAGGGATATTTCACCGCCCATCCA    60
eCPX_TagRFP_K10_clone5_pTrcHisrev       TCAATGATGATGATGATGATGTTTTTCTACACTACGCAGGGATATTTCACCGCCCATCCA    60
eCPX_TagRFP_K10_clone6_pTrcHisrev       TCAATGATGATGATGATGATGTTTTTCTACACTACGCAGGGATATTTCACCGCCCATCCA    60
eCPX_TagRFP_K10_clone7_pTrcHisrev       TCAATGATGATGATGATGATGTTTTTCTACACTACGCAGGGATATTTCACCGCCCATCCA    60
eCPX_TagRFP_K10_clone8_pTrcHisrev       TCAATGATGATGATGATGATGTTTTTCTACACTACGCAGGGATATTTCACCGCCCATCCA    60
eCPX_TagRFP_K10_clone9_pTrcHisrev       TCAATGATGATGATGATGATGTTTTTCTACACTACGCAGGGATATTTCACCGCCCATCCA    60
eCPX_TagRFP_K10_clone10_pTrcHisrev      TCAATGATGATGATGATGATGTTTTTCTACACTACGCAGGGATATTTCACCGCCCATCCA    60
BirA_rev                                TCAATGATGATGATGATGATGTTTTTCTGCACTACGCAGGGATATTTCACCGCCCATCCA    60
                                        **************************** *******************************
```

```
eCPX_TagRFP_K10_clone1_pTrcHisrev       GGGTTTTATTATTCCATCCTGCTCAAGTAATAAAGCCCCCAGTTTGTCTATTCCGCGTGA    120
eCPX_TagRFP_K10_clone2_pTrcHisrev       GGGTTTTATTATTCCATCCTGCTCAAGTAATAAAGCCCCCAGTTTGTCTATTCCGCGTGA    120
eCPX_TagRFP_K10_clone3_pTrcHisrev       GGGTTTTATTATTCCATCCTGCTCAAGTAATAAAGCCCCCAGTTTGTCTATTCCGCGTGA    120
eCPX_TagRFP_K10_clone4_pTrcHisrev       GGGTTTTATTATTCCATCCTGCTCAAGTAATAAAGCCCCCAGTTTGTCTATTCCGCGTGA    120
eCPX_TagRFP_K10_clone5_pTrcHisrev       GGGTTTTATTATTCCATCCTGCTCAAGTAATAAAGCCCCCAGTTTGTCTATTCCGCGTGA    120
eCPX_TagRFP_K10_clone6_pTrcHisrev       GGGTTTTATTATTCCATCCTGCTCAAGTAATAAAGCCCCCAGTTTGTCTATTCCGCGTGA    120
eCPX_TagRFP_K10_clone7_pTrcHisrev       GGGTTTTATTATTCCATCCTGCTCAAGTAATAAAGCCCCCAGTTTGTCTATTCCGCGTGA    120
eCPX_TagRFP_K10_clone8_pTrcHisrev       GGGTTTTATTATTCCATCCTGCTCAAGTAATAAAGCCCCCAGTTTGTCTATTCCGCGTGA    120
eCPX_TagRFP_K10_clone9_pTrcHisrev       GGGTTTTATTATTCCATCCTGCTCAAGTAATAAAGCCCCCAGTTTGTCTATTCCGCGTGA    120
eCPX_TagRFP_K10_clone10_pTrcHisrev      GGGTTTTATTATTCCATCCTGCTCAAGTAATAAAGCCCCCAGTTTGTCTATTCCGCGTGA    120
BirA_rev                                GGGTTTTATTATTCCATCCTGCTCAAGTAATAAAGCCCCCTGTTTGTCTATTCCGCGTGA    120
                                        **************************************** *******************

eCPX_TagRFP_K10_clone1_pTrcHisrev       AATGCCAAATATTTCTTTATCACCAATGATAAGTTTCACTGGGCGATTAATAGAATTATC    180
eCPX_TagRFP_K10_clone2_pTrcHisrev       AATGCCAAATATTTCTTTATCACCAATGATAAGTTTCACTGGGCGATTAATAGAATTATC    180
eCPX_TagRFP_K10_clone3_pTrcHisrev       AATGCCAAATATTTCTTTATCACCAATGATAAGTTTCACTGGGCGATTAATAGAATTATC    180
eCPX_TagRFP_K10_clone4_pTrcHisrev       AATGCCAAATATTTCTTTATCACCAATGATAAGTTTCACTGGGCGATTAATAGAATTATC    180
eCPX_TagRFP_K10_clone5_pTrcHisrev       AATGCCAAATATTTCTTTATCACCAATGATAAGTTTCACTGGGCGATTAATAGAATTATC    180
eCPX_TagRFP_K10_clone6_pTrcHisrev       AATGCCAAATATTTCTTTATCACCAATGATAAGTTTCACTGGGCGATTAATAGAATTATC    180
eCPX_TagRFP_K10_clone7_pTrcHisrev       AATGCCAAATATTTCTTTATCACCAATGATAAGTTTCACTGGGCGATTAATAGAATTATC    180
eCPX_TagRFP_K10_clone8_pTrcHisrev       AATGCCAAATATTTCTTTATCACCAATGATAAGTTTCACTGGGCGATTAATAGAATTATC    180
eCPX_TagRFP_K10_clone9_pTrcHisrev       AATGCCAAATATTTCTTTATCACCAATGATAAGTTTCACTGGGCGATTAATAGAATTATC    180
eCPX_TagRFP_K10_clone10_pTrcHisrev      AATGCCAAATATTTCTTTATCACCAATGATAAGTTTCACTGGGCGATTAATAGAATTATC    180
BirA_rev                                AATGCCAAATATTTCTTTATCACCAATGATAAGTTTCACTGGGCGATTAATAAAATTATC    180
                                        **************************************************** *******

eCPX_TagRFP_K10_clone1_pTrcHisrev       CAGCTTTTCCCAGCGCGACAGATAAGGTGCCAATCCTTCTTGTTCGAAGAGTTCCAACGC    240
eCPX_TagRFP_K10_clone2_pTrcHisrev       CAGCTTTTCCCAGCGCGACAGATAAGGTGCCAATCCTTCTTGTTCGAAGAGTTCCAACGC    240
eCPX_TagRFP_K10_clone3_pTrcHisrev       CAGCTTTTCCCAGCGCGACAGATAAGGTGCCAATCCTTCTTGTTCGAAGAGTTCCAACGC    240
eCPX_TagRFP_K10_clone4_pTrcHisrev       CAGCTTTTCCCAGCGCGACAGATAAGGTGCCAATCCTTCTTGTTCGAAGAGTTCCAACGC    240
eCPX_TagRFP_K10_clone5_pTrcHisrev       CAGCTTTTCCCAGCGCGACAGATAAGGTGCCAATCCTTCTTGTTCGAAGAGTTCCAACGC    240
eCPX_TagRFP_K10_clone6_pTrcHisrev       CAGCTTTTCCCAGCGCGACAGATAAGGTGCCAATCCTTCTTGTTCGAAGAGTTCCAACGC    240
eCPX_TagRFP_K10_clone7_pTrcHisrev       CAGCTTTTCCCAGCGCGACAGATAAGGTGCCAATCCTTCTTGTTCGAAGAGTTCCAACGC    240
eCPX_TagRFP_K10_clone8_pTrcHisrev       CAGCTTTTCCCAGCGCGACAGATAAGGTGCCAATCCTTCTTGTTCGAAGAGTTCCAACGC    240
eCPX_TagRFP_K10_clone9_pTrcHisrev       CAGCTTTTCCCAGCGCGACAGATAAGGTGCCAATCCTTCTTGTTCGAAGAGTTCCAACGC    240
eCPX_TagRFP_K10_clone10_pTrcHisrev      CAGCTTTTCCCAGCGCGACAGATAAGGTGCCAATCCTTCTTGTTCGAAGAGTTCCAACGC    240
BirA_rev                                CAGCTTTTCCCAGCGCGACAGATAAGGTGCCAATCCTTCTTGTTCGAAGAGTTCCAACGC    240
                                        ************************************************************

eCPX_TagRFP_K10_clone1_pTrcHisrev       AGCACGTAATTCACGTATTAGCATGGCCGCCAACGTATTACGATCGAGATTGATCCCCGC    300
eCPX_TagRFP_K10_clone2_pTrcHisrev       AGCACGTAATTCACGTATTAGCATGGCCGCCAACGTATTACGATCGAGATTGATCCCCGC    300
eCPX_TagRFP_K10_clone3_pTrcHisrev       AGCACGTAATTCACGTATTAGCATGGCCGCCAACGTATTACGATCGAGATTGATCCCCGC    300
eCPX_TagRFP_K10_clone4_pTrcHisrev       AGCACGTAATTCACGTATTAGCATGGCCGCCAACGTATTACGATCGAGATTGATCCCCGC    300
eCPX_TagRFP_K10_clone5_pTrcHisrev       AGCACGTAATTCACGTATTAGCATGGCCGCCAACGTATTACGATCGAGATTGATCCCCGC    300
eCPX_TagRFP_K10_clone6_pTrcHisrev       AGCACGTAATTCACGTATTAGCATGGCCGCCAACGTATTACGATCGAGATTGATCCCCGC    300
eCPX_TagRFP_K10_clone7_pTrcHisrev       AGCACGTAATTCACGTATTAGCATGGCCGCCAACGTATTACGATCGAGATTGATCCCCGC    300
eCPX_TagRFP_K10_clone8_pTrcHisrev       AGCACGTAATTCACGTATTAGCATGGCCGCCAACGTATTACGATCGAGATTGATCCCCGC    300
eCPX_TagRFP_K10_clone9_pTrcHisrev       AGCACGTAATTCACGTATTAGCATGGCCGCCAACGTATTACGATCGAGATTGATCCCCGC    300
eCPX_TagRFP_K10_clone10_pTrcHisrev      AGCACGTAATTCACGTATTAGCATGGCCGCCAACGTATTACGATCGAGATTGATCCCCGC    300
BirA_rev                                AGCACGTAATTCACGTATTAGCATGGCCGCCAACGTATTACGATCGAGATTGATCCCCGC    300
                                        ************************************************************
```

```
eCPX_TagRFP_K10_clone1_pTrcHisrev       TTCCTGCAGCGTGATCCACCCCTGATTAACGACACTCTCTTCAACACGGCGCATTGCCAT    360
eCPX_TagRFP_K10_clone2_pTrcHisrev       TTCCTGCAGCGTGATCCACCCCTGATTAACGACACTCTCTTCAACACGGCGCATTGCCAT    360
eCPX_TagRFP_K10_clone3_pTrcHisrev       TTCCTGCAGCGTGATCCACCCCTGATTAACGACACTCTCTTCAACACGGCGCATTGCCAT    360
eCPX_TagRFP_K10_clone4_pTrcHisrev       TTCCTGCAGCGTGATCCACCCCTGATTAACGACACTCTCTTCAACACGGCGCATTGCCAT    360
eCPX_TagRFP_K10_clone5_pTrcHisrev       TTCCTGCAGCGTGATCCACCCCTGATTAACGACACTCTCTTCAACACGGCGCATTGCCAT    360
eCPX_TagRFP_K10_clone6_pTrcHisrev       TTCCTGCAGCGTGATCCACCCCTGATTAACGACACTCTCTTCAACACGGCGCATTGCCAT    360
eCPX_TagRFP_K10_clone7_pTrcHisrev       TTCCTGCAGCGTGATCCACCCCTGATTAACGACACTCTCTTCAACACGGCGCATTGCCAT    360
eCPX_TagRFP_K10_clone8_pTrcHisrev       TTCCTGCAGCGTGATCCACCCCTGATTAACGACACTCTCTTCAACACGGCGCATTGCCAT    360
eCPX_TagRFP_K10_clone9_pTrcHisrev       TTCCTGCAGCGTGATCCACCCCTGATTAACGACACTCTCTTCAACACGGCGCATTGCCAT    360
eCPX_TagRFP_K10_clone10_pTrcHisrev      TTCCTGCAGCGTGATCCACCCCTGATTAACGACACTCTCTTCAACACGGCGCATTGCCAT    360
BirA_rev                                TTCCTGCAGCGTGATCCACCCCTGATTAACGACACTCTCTTCAACACGGCGCATTGCCAT    360
                                        ************************************************************

eCPX_TagRFP_K10_clone1_pTrcHisrev       GTTGATCCCGGCTCCAATGACTATTTGCGCCGCATCGCCAGTTTTGCCCGTCAGCTCCAC    420
eCPX_TagRFP_K10_clone2_pTrcHisrev       GTTGATCCCGGCTCCAATGACTATTTGCGCCGCATCGCCAGTTTTGCCCGTCAGCTCCAC    420
eCPX_TagRFP_K10_clone3_pTrcHisrev       GTTGATCCCGGCTCCAATGACTATTTGCGCCGCATCGCCAGTTTTGCCCGTCAGCTCCAC    420
eCPX_TagRFP_K10_clone4_pTrcHisrev       GTTGATCCCGGCTCCAATGACTATTTGCGCCGCATCGCCAGTTTTGCCCGTCAGCTCCAC    420
eCPX_TagRFP_K10_clone5_pTrcHisrev       GTTGATCCCGGCTCCAATGACTATTTGCGCCGCATCGCCAGTTTTGCCCGTCAGCTCCAC    420
eCPX_TagRFP_K10_clone6_pTrcHisrev       GTTGATCCCGGCTCCAATGACTATTTGCGCCGCATCGCCAGTTTTGCCCGTCAGCTCCAC    420
eCPX_TagRFP_K10_clone7_pTrcHisrev       GTTGATCCCGGCTCCAATGACTATTTGCGCCGCATCGCCAGTTTTGCCCGTCAGCTCCAC    420
eCPX_TagRFP_K10_clone8_pTrcHisrev       GTTGATCCCGGCTCCAATGACTATTTGCGCCGCATCGCCAGTTTTGCCCGTCAGCTCCAC    420
eCPX_TagRFP_K10_clone9_pTrcHisrev       GTTGATCCCGGCTCCAATGACTATTTGCGCCGCATCGCCAGTTTTGCCCGTCAGCTCCAC    420
eCPX_TagRFP_K10_clone10_pTrcHisrev      GTTGATCCCGGCTCCAATGACTATTTGCGCCGCATCGCCAGTTTTGCCCGTCAGCTCCAC    420
BirA_rev                                GTTGATCCCGGCTCCAATGACTATTTGCGCCGCATCGCCAGTTTTGCCAGTCAGCTCCAC    420
                                        ************************************************ ***********

eCPX_TagRFP_K10_clone1_pTrcHisrev       TAGAATGCCTGCCAGCTTGCGATCCTGCAGATAGAGGTCATTAGGCCATTTAACACGAAC    480
eCPX_TagRFP_K10_clone2_pTrcHisrev       TAGAATGCCTGCCAGCTTGCGATCCTGCAGATAGAGGTCATTAGGCCATTTAACACGAAC    480
eCPX_TagRFP_K10_clone3_pTrcHisrev       TAGAATGCCTGCCAGCTTGCGATCCTGCAGATAGAGGTCATTAGGCCATTTAACACGAAC    480
eCPX_TagRFP_K10_clone4_pTrcHisrev       TAGAATGCCTGCCAGCTTGCGATCCTGCAGATAGAGGTCATTAGGCCATTTAACACGAAC    480
eCPX_TagRFP_K10_clone5_pTrcHisrev       TAGAATGCCTGCCAGCTTGCGATCCTGCAGATAGAGGTCATTAGGCCATTTAACACGAAC    480
eCPX_TagRFP_K10_clone6_pTrcHisrev       TAGAATGCCTGCCAGCTTGCGATCCTGCAGATAGAGGTCATTAGGCCATTTAACACGAAC    480
eCPX_TagRFP_K10_clone7_pTrcHisrev       TAGAATGCCTGCCAGCTTGCGATCCTGCAGATAGAGGTCATTAGGCCATTTAACACGAAC    480
eCPX_TagRFP_K10_clone8_pTrcHisrev       TAGAATGCCTGCCAGCTTGCGATCCTGCAGATAGAGGTCATTAGGCCATTTAACACGAAC    480
eCPX_TagRFP_K10_clone9_pTrcHisrev       TAGAATGCCTGCCAGCTTGCGATCCTGCAGATAGAGGTCATTAGGCCATTTAACACGAAC    480
eCPX_TagRFP_K10_clone10_pTrcHisrev      TAGAATGCCTGCCAGCTTGCGATCCTGCAGATAGAGGTCATTAGGCCATTTAACACGAAC    480
BirA_rev                                CAGAATGCCTGCCAGCTTGCGATCCTGCAGATAGAGGTCATTAGGCCATTTAACACGAAC    480
                                         ***********************************************************

eCPX_TagRFP_K10_clone1_pTrcHisrev       TTTATCTGCACC    492
eCPX_TagRFP_K10_clone2_pTrcHisrev       TTTATCTGCACC    492
eCPX_TagRFP_K10_clone3_pTrcHisrev       TTTATCTGCACC    492
eCPX_TagRFP_K10_clone4_pTrcHisrev       TTTATCTGCACC    492
eCPX_TagRFP_K10_clone5_pTrcHisrev       TTTATCTGCACC    492
eCPX_TagRFP_K10_clone6_pTrcHisrev       TTTATCTGCACC    492
eCPX_TagRFP_K10_clone7_pTrcHisrev       TTTATCTGCACC    492
eCPX_TagRFP_K10_clone8_pTrcHisrev       TTTATCTGCACC    492
eCPX_TagRFP_K10_clone9_pTrcHisrev       TTTATCTGCACC    492
eCPX_TagRFP_K10_clone10_pTrcHisrev      TTTATCTGCACC    492
BirA_rev                                TTTATCTGCACC    492
                                        ************
```

(b) Alignment of BirA-6xHis and 12 isolated γENaC K190 clones

```
CLUSTAL O(1.2.4) multiple sequence alignment


190_2           TCAATGATGATGATGATGATGTTTTTCTGCACTACGCAGGGATATTTCACCGCCCATCCA    60
190_9           TCAATGATGATGATGATGATGTTTTTCTGCACTACGCAGGGATATTTCACCGCCCATCCA    60
190_1           TCAATGATGATGATGATGATGTTTTTCTGCACTACGCAGGGATGTTCCACCGCCCATCCA    60
190_3           TCAATGATGATGATGATGATGTTTTTCTGCACTACGCAGGGATGTTCCACCGCCCATCCA    60
190_4           TCAATGATGATGATGATGATGTTTTTCTGCACTACGCAGGGATGTTCCACCGCCCATCCA    60
190_5           TCAATGATGATGATGATGATGTTTTTCTGCACTACGCAGGGATGTTCCACCGCCCATCCA    60
190_6           TCAATGATGATGATGATGATGTTTTTCTGCACTACGCAGGGATGTTCCACCGCCCATCCA    60
190_7           TCAATGATGATGATGATGATGTTTTTCTGCACTACGCAGGGATGTTCCACCGCCCATCCA    60
190_8           TCAATGATGATGATGATGATGTTTTTCTGCACTACGCAGGGATGTTCCACCGCCCATCCA    60
190_10          TCAATGATGATGATGATGATGTTTTTCTGCACTACGCAGGGATGTTCCACCGCCCATCCA    60
190_12          TCAATGATGATGATGATGATGTTTTTCTGCACTACGCAGGGATGTTCCACCGCCCATCCA    60
190_11          TCAATGATGATGATGATGATGTTTTTCTGCACTACGCAGGGATATTTCACCGCCCATCCA    60
BirA_rev        TCAATGATGATGATGATGATGTTTTTCTGCACTACGCAGGGATATTTCACCGCCCATCCA    60
                ******************************************* ** *************
```

```
190_2       GGGTTTATTATTCCATCCTGCTCAAGTAATAAAGCCCCCTGTTTGCCTATTCCGCGTGA   120
190_9       GGGTTTATTATTCCATCCTGCTCAAGTAATAAAGCCCCCTGTTTGCCTATTCCGCGTGA   120
190_1       GGGTTTATTATTCCATCCTGCTCAAGTAATAAAGCCCCCTGTTTGTCTATTCCGCGTGA   120
190_3       GGGTTTATTATTCCATCCTGCTCAAGTAATAAAGCCCCCTGTTTGTCTATTCCGCGTGA   120
190_4       GGGTTTATTATTCCATCCTGCTCAAGTAATAAAGCCCCCTGTTTGTCTATTCCGCGTGA   120
190_5       GGGTTTATTATTCCATCCTGCTCAAGTAATAAAGCCCCCTGTTTGTCTATTCCGCGTGA   120
190_6       GGGTTTATTATTCCATCCTGCTCAAGTAATAAAGCCCCCTGTTTGTCTATTCCGCGTGA   120
190_7       GGGTTTATTATTCCATCCTGCTCAAGTAATAAAGCCCCCTGTTTGTCTATTCCGCGTGA   120
190_8       GGGTTTATTATTCCATCCTGCTCAAGTAATAAAGCCCCCTGTTTGTCTATTCCGCGTGA   120
190_10      GGGTTTATTATTCCATCCTGCTCAAGTAATAAAGCCCCCTGTTTGTCTATTCCGCGTGA   120
190_12      GGGTTTATTATTCCATCCTGCTCAAGTAATAAAGCCCCCTGTTTGTCTATTCCGCGTGA   120
190_11      TGGTTTATTATTCCATCCTGCTCAAGTAATAAAGCCCCCTGTTTGTCTATTCCGCGTGA   120
BirA_rev    GGGTTTATTATTCCATCCTGCTCAAGTAATAAAGCCCCCTGTTTGTCTATTCCGCGTGA   120
             ******************************************** **************

190_2       AATGCCAAATATTTCTTATCACCAATGATAAGTTTCACTGGGCGATTAATAAAATTATC   180
190_9       AATGCCAAATATTTCTTATCACCAATGATAAGTTTCACTGGGCGATTAATAAAATTATC   180
190_1       AATGCTAAATATTTCTTATCACCAATGTTAAGTTTCACTGGGCGATTAATAAAATTATC   180
190_3       AATGCTAAATATTTCTTATCACCAATGTTAAGTTTCACTGGGCGATTAATAAAATTATC   180
190_4       AATGCTAAATATTTCTTATCACCAATGTTAAGTTTCACTGGGCGATTAATAAAATTATC   180
190_5       AATGCTAAATATTTCTTATCACCAATGTTAAGTTTCACTGGGCGATTAATAAAATTATC   180
190_6       AATGCTAAATATTTCTTATCACCAATGTTAAGTTTCACTGGGCGATTAATAAAATTATC   180
190_7       AATGCTAAATATTTCTTATCACCAATGTTAAGTTTCACTGGGCGATTAATAAAATTATC   180
190_8       AATGCTAAATATTTCTTATCACCAATGTTAAGTTTCACTGGGCGATTAATAAAATTATC   180
190_10      AATGCTAAATATTTCTTATCACCAATGTTAAGTTTCACTGGGCGATTAATAAAATTATC   180
190_12      AATGCTAAATATTTCTTATCACCAATGTTAAGTTTCACTGGGCGATTAATAAAATTATC   180
190_11      AATGCCAAATATTTCTTATCACCAATGATAAGTTTCACTGGGCGATTAATAAAATTATC   180
BirA_rev    AATGCCAAATATTTCTTATCACCAATGATAAGTTTCACTGGGCGATTAATAAAATTATC   180
            ***** ********************* ********************************

190_2       CAGCTTTTCCCAGCGCGACAGATAAGGTGCCAATCCTTCCTGTTGGAAGAGTTCCAACGC   240
190_9       CAGCTTTTCCCAGCGCGACAGATAAGGTGCCAATCCTTCCTGTTGGAAGAGTTCCAACGC   240
190_1       CAGCTTTTCCCAGCGCGTCAGATAAGGTGCCAATCCTTCTCGTTCGAAGAGTTCCAACGC   240
190_3       CAGCTTTTCCCAGCGCGTCAGATAAGGTGCCAATCCTTCTCGTTCGAAGAGTTCCAACGC   240
190_4       CAGCTTTTCCCAGCGCGTCAGATAAGGTGCCAATCCTTCTCGTTCGAAGAGTTCCAACGC   240
190_5       CAGCTTTTCCCAGCGCGTCAGATAAGGTGCCAATCCTTCTCGTTCGAAGAGTTCCAACGC   240
190_6       CAGCTTTTCCCAGCGCGTCAGATAAGGTGCCAATCCTTCTCGTTCGAAGAGTTCCAACGC   240
190_7       CAGCTTTTCCCAGCGCGTCAGATAAGGTGCCAATCCTTCTCGTTCGAAGAGTTCCAACGC   240
190_8       CAGCTTTTCCCAGCGCGTCAGATAAGGTGCCAATCCTTCTCGTTCGAAGAGTTCCAACGC   240
190_10      CAGCTTTTCCCAGCGCGTCAGATAAGGTGCCAATCCTTCTCGTTCGAAGAGTTCCAACGC   240
190_12      CAGCTTTTCCCAGCGCGTCAGATAAGGTGCCAATCCTTCTCGTTCGAAGAGTTCCAACGC   240
190_11      CAGCTTTTCCCAGCGCGACAGATAAGGTGCCAATCCTTCTTGTTCGAAGAGTTCCAACGC   240
BirA_rev    CAGCTTTTCCCAGCGCGACAGATAAGGTGCCAATCCTTCTTGTTCGAAGAGTTCCAACGC   240
            ***************** *********************  *** ***************
```

```
190_2           AGCACGTAATTCACGTATTAGCATGGCCGCCAACGTATTACGATCGAGATTGATCCCCGC    300
190_9           AGCACGTAATTCACGTATTAGCATGGCCGCCAACGTATTACGATCGAGATTGATCCCCGC    300
190_1           AGCACGTAATTCACGTATTAGCATGGCCGCCAACGTATTACGATCGAGATTGATCCCCGC    300
190_3           AGCACGTAATTCACGTATTAGCATGGCCGCCAACGTATTACGATCGAGATTGATCCCCGC    300
190_4           AGCACGTAATTCACGTATTAGCATGGCCGCCAACGTATTACGATCGAGATTGATCCCCGC    300
190_5           AGCACGTAATTCACGTATTAGCATGGCCGCCAACGTATTACGATCGAGATTGATCCCCGC    300
190_6           AGCACGTAATTCACGTATTAGCATGGCCGCCAACGTATTACGATCGAGATTGATCCCCGC    300
190_7           AGCACGTAATTCACGTATTAGCATGGCCGCCAACGTATTACGATCGAGATTGATCCCCGC    300
190_8           AGCACGTAATTCACGTATTAGCATGGCCGCCAACGTATTACGATCGAGATTGATCCCCGC    300
190_10          AGCACGTAATTCACGTATTAGCATGGCCGCCAACGTATTACGATCGAGATTGATCCCCGC    300
190_12          AGCACGTAATTCACGTATTAGCATGGCCGCCAACGTATTACGATCGAGATTGATCCCCGC    300
190_11          AGCACGTAATTCACGTATTAGCATGGCCGCCAACGTATTACGATCGAGATTGAACCCCGC    300
BirA_rev        AGCACGTAATTCACGTATTAGCATGGCCGCCAACGTATTACGATCGAGATTGATCCCCGC    300
                **************************************************** *******

190_2           TTCCTGCAGGGTGATCCACCCCTGATTAACGACACTCTCTTCAACACGGCGCATTGCCAT    360
190_9           TTCCTGCAGGGTGATCCACCCCTGATTAACGACACTCTCTTCAACACGGCGCATTGCCAT    360
190_1           TTCCTGCAGCGTGATCCACCCCTGATTAACGACACTCTCTTCAACACGGCGCATTGCCAT    360
190_3           TTCCTGCAGCGTGATCCACCCCTGATTAACGACACTCTCTTCAACACGGCGCATTGCCAT    360
190_4           TTCCTGCAGCGTGATCCACCCCTGATTAACGACACTCTCTTCAACACGGCGCATTGCCAT    360
190_5           TTCCTGCAGCGTGATCCACCCCTGATTAACGACACTCTCTTCAACACGGCGCATTGCCAT    360
190_6           TTCCTGCAGCGTGATCCACCCCTGATTAACGACACTCTCTTCAACACGGCGCATTGCCAT    360
190_7           TTCCTGCAGCGTGATCCACCCCTGATTAACGACACTCTCTTCAACACGGCGCATTGCCAT    360
190_8           TTCCTGCAGCGTGATCCACCCCTGATTAACGACACTCTCTTCAACACGGCGCATTGCCAT    360
190_10          TTCCTGCAGCGTGATCCACCCCTGATTAACGACACTCTCTTCAACACGGCGCATTGCCAT    360
190_12          TTCCTGCAGCGTGATCCACCCCTGATTAACGACACTCTCTTCAACACGGCGCATTGCCAT    360
190_11          TTCCTGCAGCGTGATCCACCCCTGATTAACGACACTCTCTTCAACACGGCGCATTGCCAT    360
BirA_rev        TTCCTGCAGCGTGATCCACCCCTGATTAACGACACTCTCTTCAACACGGCGCATTGCCAT    360
                ********* **************************************************

190_2           GTTGATCCCTGCTCCAATGACTATTTGCGCCGCATCGCCAGTTTTGCCAGTCAGCTCAAC    420
190_9           GTTGATCCCTGCTCCAATGACTATTTGCGCCGCATCGCCAGTTTTGCCAGTCAGCTCAAC    420
190_1           GTTAATCCCGGCTCCAATGACTATTTGCGCCGCATCGCCAGTTTTGCCAGTCAGCTCCAC    420
190_3           GTTAATCCCGGCTCCAATGACTATTTGCGCCGCATCGCCAGTTTTGCCAGTCAGCTCCAC    420
190_4           GTTAATCCCGGCTCCAATGACTATTTGCGCCGCATCGCCAGTTTTGCCAGTCAGCTCCAC    420
190_5           GTTAATCCCGGCTCCAATGACTATTTGCGCCGCATCGCCAGTTTTGCCAGTCAGCTCCAC    420
190_6           GTTAATCCCGGCTCCAATGACTATTTGCGCCGCATCGCCAGTTTTGCCAGTCAGCTCCAC    420
190_7           GTTAATCCCGGCTCCAATGACTATTTGCGCCGCATCGCCAGTTTTGCCAGTCAGCTCCAC    420
190_8           GTTAATCCCGGCTCCAATGACTATTTGCGCCGCATCGCCAGTTTTGCCAGTCAGCTCCAC    420
190_10          GTTAATCCCGGCTCCAATGACTATTTGCGCCGCATCGCCAGTTTTGCCAGTCAGCTCCAC    420
190_12          GTTAATCCCGGCTCCAATGACTATTTGCGCCGCATCGCCAGTTTTGCCAGTCAGCTCCAC    420
190_11          GTTGATCCCGACTCCAATGACTATTTGCGCCGCATCGCCAGTTTGGCCAGTCAGCTCCAC    420
BirA_rev        GTTGATCCCGGCTCCAATGACTATTTGCGCCGCATCGCCAGTTTTGCCAGTCAGCTCCAC    420
                *** *****  ********************************* ************ **
```

```
190_2       CAGAATGCCTGCCAGCTTGCGATCCTGCAGATAGAAGTCATCAGGCCGTTTAACACGAAC    480
190_9       CAGAATGCCTGCCAGCTTGCGATCCTGCAGATAGAAGTCATCAGGCCGTTTAACACGAAC    480
190_1       CAGAATGCCTGCCAGCTTGCGATCCTGCAGATAGAGGTCATTAGGCCATTTAACACGAAC    480
190_3       CAGAATGCCTGCCAGCTTGCGATCCTGCAGATAGAGGTCATTAGGCCATTTAACACGAAC    480
190_4       CAGAATGCCTGCCAGCTTGCGATCCTGCAGATAGAGGTCATTAGGCCATTTAACACGAAC    480
190_5       CAGAATGCCTGCCAGCTTGCGATCCTGCAGATAGAGGTCATTAGGCCATTTAACACGAAC    480
190_6       CAGAATGCCTGCCAGCTTGCGATCCTGCAGATAGAGGTCATTAGGCCATTTAACACGAAC    480
190_7       CAGAATGCCTGCCAGCTTGCGATCCTGCAGATAGAGGTCATTAGGCCATTTAACACGAAC    480
190_8       CAGAATGCCTGCCAGCTTGCGATCCTGCAGATAGAGGTCATTAGGCCATTTAACACGAAC    480
190_10      CAGAATGCCTGCCAGCTTGCGATCCTGCAGATAGAGGTCATTAGGCCATTTAACACGAAC    480
190_12      CAGAATGCCTGCCAGCTTGCGATCCTGCAGATAGAGGTCATTAGGCCATTTAACACGAAC    480
190_11      CAGAATGCCTGCCAGCTTGCGATCCTGCAGATAGAGGTCATTAGGCCATTTAACACGAAC    480
BirA_rev    CAGAATGCCTGCCAGCTTGCGATCCTGCAGATAGAGGTCATTAGGCCATTTAACACGAAC    480
            *********************************** ***** ***** ************

190_2       TTTATCTGCACC    492
190_9       TTTATCTGCACC    492
190_1       TTTATCTGCACC    492
190_3       TTTATCTGCACC    492
190_4       TTTATCTGCACC    492
190_5       TTTATCTGCACC    492
190_6       TTTATCTGCACC    492
190_7       TTTATCTGCACC    492
190_8       TTTATCTGCACC    492
190_10      TTTATCTGCACC    492
190_12      TTTATCTGCACC    492
190_11      TTTATCTGCACC    492
BirA_rev    TTTATCTGCACC    492
            ************
```

(**a,b**) Multiple Sequence Alignment of selected BirA variants and BirA-6xHis using Clustal Omega (https://www.ebi.ac.uk/Tools/msa/clustalo/). The DNA sequences were obtained by sequencing in the reverse direction using primer pTrcHis rev (CTTCTGCGTTCTGATTTA ATCTG) on the isolated clones.

Supplementary Figure 11

a

>BirA-6xHis
MKDNTVPLKLIALLANGEFHSGEQLGETLGMSRAAINKHIQTLRDWGVDVFTVPGKGYSL
PEPIQLLNAKQILGQLDGGSVAVLPVIDSTNQYLLDRIGELKSGDACIAEYQQAGRGRRGR
KWFSPFGANLYLSMFWRLEQGPAAAIGLSLVIGIVMAEVLRKLGADKVRVKWPNDLYLQ
DRKLAGILVELTGKTGDAAQIVIGAGINMAMRRVEESVVNQGWITLQEAGINLDRNTLAA
MLIRELRAALELFEQEGLAPYLSRWEKLDNFINRPVKLIIGDKEIFGISRGIDKQGALLLEQD
GIIKPWMGGEISLRSAEKHHHHHH
>TagRFP_K10A

MKDNTVPLKLIALLANGEFHSGEQLGETLGMSRAAINKHTQTLRDWGVDVFTVPGKGYSL
PEPIQLLNAKQILGQLDGGSVAVLPVIDSTNQYLLDRIGELKSGDACIAEYQQAGRGRRGR
KWFSPFGANLYLSMFWRLEQGPAAAIGLSLVIGIVMAEVLRKLGADKVRVKWPNDLYLQ
DRKLAGILVELTGKTGDAAQIVIGAGINMAMRRVEESVVNQGWITLQEAGINLDRNTLAA
MLIRELRAALELFEQEGLAPYLSRWEKLDNSINRPVKLIIGDKEIFGISRGIDKLGALLLEQD
GIIKPWMGGEISLRSVEKHHHHHH
>TagRFP_K231,235
MKDNTVPLKLIALLANGEFHSGEQSGETLGMSRAAINKHIQTLRDWGVEVFTVPGKGYSL
PEPIQLLNAKQILGQLDGGSVAVLPMIDSTNQYLLDRIGELKSGDACIAEYQQAGRGRQGR
KWFSPFGANLYLSMFWRLEQGPAAAIGLSLVIGIVMAEVLRKLGADKVRVKWPNDLYLQ
DRKLAGILVELTGKTGDAAQIVIGAGINMAMRRVEESVVNQGWITLQEAGINLDRNTLAA
MLIRELRAALELCEQEGLAPYLSRWEKLDNYINRPVKLIIGDKEIFGISRGIDKQGALLLEQD
GIIKPWMGGEISLRSAEKHHHHHH

b

```
CLUSTAL O(1.2.4) multiple sequence alignment


TagRFP_K231,235      MKDNTVPLKLIALLANGEFHSGEQSGETLGMSRAAINKHIQTLRDWGVEVFTVPGKGYSL      60
BirA-6His            MKDNTVPLKLIALLANGEFHSGEQLGETLGMSRAAINKHIQTLRDWGVDVFTVPGKGYSL      60
TagRFP_K10A          MKDNTVPLKLIALLANGEFHSGEQLGETLGMSRAAINKHTQTLRDWGVDVFTVPGKGYSL      60
                     ************************ ************** ********:***********

TagRFP_K231,235      PEPIQLLNAKQILGQLDGGSVAVLPMIDSTNQYLLDRIGELKSGDACIAEYQQAGRGRQG      120
BirA-6His            PEPIQLLNAKQILGQLDGGSVAVLPVIDSTNQYLLDRIGELKSGDACIAEYQQAGRGRRG      120
TagRFP_K10A          PEPIQLLNAKQILGQLDGGSVAVLPVIDSTNQYLLDRIGELKSGDACIAEYQQAGRGRRG      120
                     *************************:********************************:*

TagRFP_K231,235      RKWFSPFGANLYLSMFWRLEQGPAAAIGLSLVIGIVMAEVLRKLGADKVRVKWPNDLYLQ      180
BirA-6His            RKWFSPFGANLYLSMFWRLEQGPAAAIGLSLVIGIVMAEVLRKLGADKVRVKWPNDLYLQ      180
TagRFP_K10A          RKWFSPFGANLYLSMFWRLEQGPAAAIGLSLVIGIVMAEVLRKLGADKVRVKWPNDLYLQ      180
                     ************************************************************

TagRFP_K231,235      DRKLAGILVELTGKTGDAAQIVIGAGINMAMRRVEESVVNQGWITLQEAGINLDRNTLAA      240
BirA-6His            DRKLAGILVELTGKTGDAAQIVIGAGINMAMRRVEESVVNQGWITLQEAGINLDRNTLAA      240
TagRFP_K10A          DRKLAGILVELTGKTGDAAQIVIGAGINMAMRRVEESVVNQGWITLQEAGINLDRNTLAA      240
                     ************************************************************

TagRFP_K231,235      MLIRELRAALELCEQEGLAPYLSRWEKLDNYINRPVKLIIGDKEIFGISRGIDKQGALLL      300
BirA-6His            MLIRELRAALELFEQEGLAPYLSRWEKLDNFINRPVKLIIGDKEIFGISRGIDKQGALLL      300
TagRFP_K10A          MLIRELRAALELFEQEGLAPYLSRWEKLDNSINRPVKLIIGDKEIFGISRGIDKLGALLL      300
                     ************ ***************** *********************** *****

TagRFP_K231,235      EQDGIIKPWMGGEISLRSAEKHHHHHH        327
BirA-6His            EQDGIIKPWMGGEISLRSAEKHHHHHH        327
TagRFP_K10A          EQDGIIKPWMGGEISLRSVEKHHHHHH        327
                     ******************.********
```

(a) Full-length sequences of BirA-6xHis, BirA variants against TagRFP (K10) and TagRFP (K231, K235). The sequence of TagRFP (K231, K235) was obtained from a randomly selected clone after the 3rd and final selection round.

(b) Multiple Sequence Alignment using Clustal Omega (https://www.ebi.ac.uk/Tools/msa/clustalo/).

Supplementary Figure 12

Uncropped version of Figure 5c

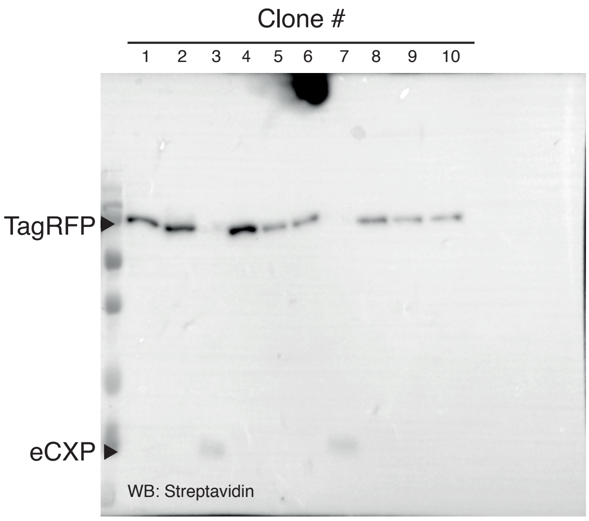

Supplementary Figure 13

```
eCPX_TagRFP_C1_pTrcHisrev               TCAATGATGATGATGATGATGTTTTTCTGCACTACGCAGGGATATTTCACCGCCCATCCA 60
eCPX_TagRFP_C8_pTrcHisrev               TCAATGATGATGATGATGATGTTTTTCTGCACTACGCAGGGATATTTCACCGCCCATCCA 60
eCPX_TagRFP_C4_pTrcHisrev               TCAATGATGATGATGATGATGTTTTTCTACACTACGCAGGGATATTTCACCGCCCATCCA 60
full_length_tagRFPK231,235_pTrcHisrev   TCAATGATGATGATGATGATGTTTTTCTGCACTACGCAGGGATATTTCACCGCCCATCCA 60
BirA_rev                                TCAATGATGATGATGATGATGTTTTTCTGCACTACGCAGGGATATTTCACCGCCCATCCA 60
                                        **************************** *******************************

eCPX_TagRFP_C1_pTrcHisrev               GGGTTTATTATTCCATCCTGCTCAAGTAATAAAGCCCCTGTTTGTCTATTCCGCGTGA 120
eCPX_TagRFP_C8_pTrcHisrev               GGGTTTATTATTCCATCCTGCTCAAGTAATAAAGCCCCTGTTTGTCTATTCCGCGTGA 120
eCPX_TagRFP_C4_pTrcHisrev               GGGTTTATTATTCCATCCTGCTCAAGTAATAAAGCCCCAGTTTGTCTATTCCGCGTGA 120
full_length_tagRFPK231,235_pTrcHisrev   GGGTTTATTATTCCATCCTGCTCAAGTAATAAAGCCCCTGTTTGTCTATTCCGCGTGA 120
BirA_rev                                GGGTTTATTATTCCATCCTGCTCAAGTAATAAAGCCCCTGTTTGTCTATTCCGCGTGA 120
                                        ************************************** *********************

eCPX_TagRFP_C1_pTrcHisrev               AATGCCAAATATTTCTTTATCACCAATGATAAGTTTCACCGGGCGATTAATAAAATTATC 180
eCPX_TagRFP_C8_pTrcHisrev               AATGCCAAATATTTCTTTATCACCAATGATAAGTTTCACCGGGCGATTAATAAAATTATC 180
eCPX_TagRFP_C4_pTrcHisrev               AATGCCAAATATTTCTTTATCACCAATGATAAGTTTCACTGGGCGATTAATAGAATTATC 180
full_length_tagRFPK231,235_pTrcHisrev   AATGCCAAATATTTCTTTATCACCAATGATAAGTTTCACTGGGCGATTAATATAATTATC 180
BirA_rev                                AATGCCAAATATTTCTTTATCACCAATGATAAGTTTCACTGGGCGATTAATAAAATTATC 180
                                        *************************************** ************ *******

eCPX_TagRFP_C1_pTrcHisrev               CAGCTTTTCCCAGCGCGACAGATAAGGTGCCAATCCTTCTTGTTCGAAGAGTTCCAACGC 240
eCPX_TagRFP_C8_pTrcHisrev               CAGCTTTTCCCAGCGCGACAGATAAGGTGCCAATCCTTCTTGTTCGAAGAGTTCCAACGC 240
eCPX_TagRFP_C4_pTrcHisrev               CAGCTTTTCCCAGCGCGACAGATAAGGTGCCAATCCTTCTTGTTCGAAGAGTTCCAACGC 240
full_length_tagRFPK231,235_pTrcHisrev   CAGCTTTTCCCAGCGCGACAGATAAGGTGCCAATCCTTCATGTTCGAAGAGTTCCAACGC 240
BirA_rev                                CAGCTTTTCCCAGCGCGACAGATAAGGTGCCAATCCTTCTTGTTCGAAGAGTTCCAACGC 240
                                        *************************************** ********************

eCPX_TagRFP_C1_pTrcHisrev               AGCACGTAATTCACGTATTAGCATGGCCGCCAACGTATTACGATCGAGATTGATCCCCGC 300
eCPX_TagRFP_C8_pTrcHisrev               AGCACGTAATTCACGTATTAGCATGGCCGCCAACGTATTACGATCGAGATTGATCCCCGC 300
eCPX_TagRFP_C4_pTrcHisrev               AGCACGTAATTCACGTATTAGCATGGCCGCCAACGTATTACGATCGAGATTGATCCCCGC 300
full_length_tagRFPK231,235_pTrcHisrev   AGCACGTAATTCACGTATTAGCATGGCCGCCAACGTATTACGATCGAGATTGATCCCCGC 300
BirA_rev                                AGCACGTAATTCACGTATTAGCATGGCCGCCAACGTATTACGATCGAGATTGATCCCCGC 300
                                        ************************************************************

eCPX_TagRFP_C1_pTrcHisrev               TTCCTGCAGCGTGATCCACCCCTGATTAACGACACTCACTTCAACACGGCGCATTGCCAT 360
eCPX_TagRFP_C8_pTrcHisrev               TTCCTGCAGCGTGATCCACCCCTGATTAACGACACTCACTTCAACACGGCGCATTGCCAT 360
eCPX_TagRFP_C4_pTrcHisrev               TTCCTGCAGCGTGATCCACCCCTGATTAACGACACTCTCTTCAACACGGCGCATTGCCAT 360
full_length_tagRFPK231,235_pTrcHisrev   TTCCTGCAGCGTGATCCACCCCTGATTAACGACACTCTCTTCAACACGGCGCATTGCCAT 360
BirA_rev                                TTCCTGCAGCGTGATCCACCCCTGATTAACGACACTCTCTTCAACACGGCGCATTGCCAT 360
                                        ************************************* **********************

eCPX_TagRFP_C1_pTrcHisrev               GTTGATCCCGGCTCCAATGACTATTTGCGCCGCATCGCCAAGTTTTGCCAGTCAGCTCCA 420
eCPX_TagRFP_C8_pTrcHisrev               GTTGATCCCGGCTCCAATGACTATTTGCGCCGCATCGCCAAGTTTTGCCAGTCAGCTCCA 420
eCPX_TagRFP_C4_pTrcHisrev               GTTGATCCCGGCTCCAATGACTATTTGCGCCGCATCGCCAG-TTTTGCCCGTCAGCTCCA 419
full_length_tagRFPK231,235_pTrcHisrev   GTTGATCCCGGCTCCAATGACTATTTGCGCCGCATCGCCAG-TTTTGCCAGTCAGCTCCA 419
BirA_rev                                GTTGATCCCGGCTCCAATGACTATTTGCGCCGCATCGCCAG-TTTTGCCAGTCAGCTCCA 419
                                        ****************************************  ******* **********

eCPX_TagRFP_C1_pTrcHisrev               CCAGAATGCCTGCCAGCTTGCGATCCTGCAGATAGAGGTCATTAGGCCATTTAACACGAA 480
eCPX_TagRFP_C8_pTrcHisrev               CCAGAATGCCTGCCAGCTTGCGATCCTGCAGATAGAGGTCATTAGGCCATTTAACACGAA 480
eCPX_TagRFP_C4_pTrcHisrev               CTAGAATGCCTGCCAGCTTGCGATCCTGCAGATAGAGGTCATTAGGCCATTTAACACGAA 479
full_length_tagRFPK231,235_pTrcHisrev   CCAGAATGCCTGCCAGCTTGCGATCCTGCAGATAGAGGTCATTAGGCCATTTAACACGAA 479
BirA_rev                                CCAGAATGCCTGCCAGCTTGCGATCCTGCAGATAGAGGTCATTAGGCCATTTAACACGAA 479
                                        * **********************************************************

eCPX_TagRFP_C1_pTrcHisrev               CTTTATCTGCACC        493
eCPX_TagRFP_C8_pTrcHisrev               CTTTATCTGCACC        493
eCPX_TagRFP_C4_pTrcHisrev               CTTTATCTGCACC        492
full_length_tagRFPK231,235_pTrcHisrev   CTTTATCTGCACC        492
BirA_rev                                CTTTATCTGCACC        492
                                        *************
```

Multiple Sequence Alignment of isolated TagRFP (K231, K235) BirA variants and BirA-6xHis using Clustal Omega (https://www.ebi.ac.uk/Tools/msa/clustalo/). The DNA sequences were obtained by sequencing in the reverse direction using primer pTrcHis rev (CTTCTGCGTTCTGATTTA ATCTG) on the isolated clones.

Supplementary Figure 14

Uncropped version of Figure 5d (top)

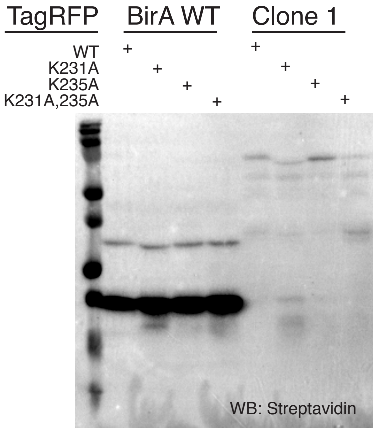

Supplementary Figure 15

Uncropped version of Figure 5d (bottom)

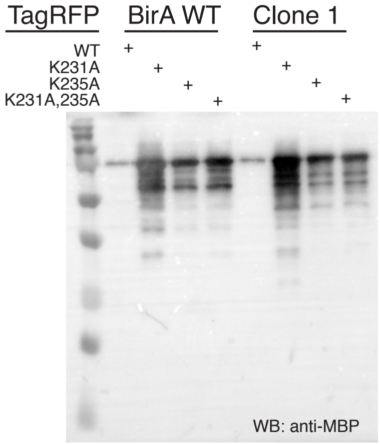

Supplement: Supplementary file 1 — Supplementary Information [file 41598_2019_40984_MOESM1_ESM.pdf]
